# A polygenic risk score analysis of psychosis endophenotypes across brain functional, structural, and cognitive domains

**DOI:** 10.1002/ajmg.b.32581

**Published:** 2017-08-29

**Authors:** Siri Ranlund, Stella Calafato, Johan H. Thygesen, Kuang Lin, Wiepke Cahn, Benedicto Crespo‐Facorro, Sonja M.C. de Zwarte, Álvaro Díez, Marta Di Forti, GROUP, Conrad Iyegbe, Assen Jablensky, Rebecca Jones, Mei‐Hua Hall, Rene Kahn, Luba Kalaydjieva, Eugenia Kravariti, Colm McDonald, Andrew M. McIntosh, Andrew McQuillin, PEIC, Marco Picchioni, Diana P. Prata, Dan Rujescu, Katja Schulze, Madiha Shaikh, Timothea Toulopoulou, Neeltje van Haren, Jim van Os, Evangelos Vassos, Muriel Walshe, WTCCC2, Cathryn Lewis, Robin M. Murray, John Powell, Elvira Bramon

**Affiliations:** ^1^ Division of Psychiatry University College London London UK; ^2^ Institute of Psychiatry Psychology and Neuroscience at King's College London and South London Maudsley NHS Foundation Trust London UK; ^3^ Nuffield Department of Population Health University of Oxford Oxford UK; ^4^ Department of Psychiatry, Brain Centre Rudolf Magnus University Medical Center Utrecht Utrecht The Netherlands; ^5^ CIBERSAM Centro Investigación Biomédica en Red Salud Mental Madrid Spain; ^6^ Department of Psychiatry, University Hospital Marqués de Valdecilla, School of Medicine University of Cantabria–IDIVAL Santander Spain; ^7^ Laboratory of Cognitive and Computational Neuroscience—Centre for Biomedical Technology (CTB) Complutense University and Technical University of Madrid Madrid Spain; ^8^ Centre for Clinical Research in Neuropsychiatry The University of Western Australia Perth, Western Australia Australia; ^9^ Psychosis Neurobiology Laboratory, Harvard Medical School McLean Hospital Belmont Massachusetts; ^10^ Harry Perkins Institute of Medical Research and Centre for Medical Research The University of Western Australia Perth Australia; ^11^ The Centre for Neuroimaging & Cognitive Genomics (NICOG) and NCBES Galway Neuroscience Centre National University of Ireland Galway Galway Ireland; ^12^ Division of Psychiatry, University of Edinburgh Royal Edinburgh Hospital Edinburgh UK; ^13^ Centre for Cognitive Ageing and Cognitive Epidemiology University of Edinburgh Edinburgh UK; ^14^ Faculdade de Medicina, Instituto de Medicina Molecular Universidade de Lisboa Portugal; ^15^ Department of Psychiatry Ludwig‐Maximilians University of Munich Munich Germany; ^16^ Department of Psychiatry, Psychotherapy and Psychosomatics University of Halle Wittenberg Halle Germany; ^17^ North East London Foundation Trust London UK; ^18^ Research Department of Clinical, Educational and Health Psychology University College London London UK; ^19^ Department of Psychology, Bilkent University Main Campus Bilkent, Ankara Turkey; ^20^ Department of Psychology The University of Hong Kong, Pokfulam Rd Hong Kong SAR China; ^21^ The State Key Laboratory of Brain and Cognitive Sciences, The University of Hong Kong The Hong Kong Jockey Club Building for Interdisciplinary Research Hong Kong SAR China; ^22^ Department of Psychiatry and Psychology, Maastricht University Medical Centre EURON Maastricht The Netherlands; ^23^ Institute of Cognitive Neuroscience University College London London UK

**Keywords:** bipolar disorder, cognition, EEG, schizophrenia, single nucleotide polymorphism (SNP)

## Abstract

This large multi‐center study investigates the relationships between genetic risk for schizophrenia and bipolar disorder, and multi‐modal endophenotypes for psychosis. The sample included 4,242 individuals; 1,087 patients with psychosis, 822 unaffected first‐degree relatives of patients, and 2,333 controls. Endophenotypes included the P300 event‐related potential (*N* = 515), lateral ventricular volume (*N* = 798), and the cognitive measures block design (*N* = 3,089), digit span (*N* = 1,437), and the Ray Auditory Verbal Learning Task (*N* = 2,406). Data were collected across 11 sites in Europe and Australia; all genotyping and genetic analyses were done at the same laboratory in the United Kingdom. We calculated polygenic risk scores for schizophrenia and bipolar disorder separately, and used linear regression to test whether polygenic scores influenced the endophenotypes. Results showed that higher polygenic scores for schizophrenia were associated with poorer performance on the block design task and explained 0.2% (*p* = 0.009) of the variance. Associations in the same direction were found for bipolar disorder scores, but this was not statistically significant at the 1% level (*p* = 0.02). The schizophrenia score explained 0.4% of variance in lateral ventricular volumes, the largest across all phenotypes examined, although this was not significant (*p* = 0.063). None of the remaining associations reached significance after correction for multiple testing (with alpha at 1%). These results indicate that common genetic variants associated with schizophrenia predict performance in spatial visualization, providing additional evidence that this measure is an endophenotype for the disorder with shared genetic risk variants. The use of endophenotypes such as this will help to characterize the effects of common genetic variation in psychosis.

## 1 INTRODUCTION

Psychotic illnesses, including schizophrenia and bipolar disorder, constitute the most severe forms of mental illnesses (WHO, 2013). They are highly heritable, with estimates of up to 80% (Cardno et al., 1999; Smoller & Finn, 2003; Sullivan, Kendler, & Neale, 2003), and there is evidence for significant genetic overlap between different psychotic diagnoses (Huang et al., 2010; Lee et al., 2013; Moskvina et al., 2009; Purcell et al., 2009; Schulze et al., 2012). Psychosis has a highly polygenic architecture, involving thousands of common single nucleotide polymorphisms (SNPs) of very small individual effects that account for an estimated 32% of the heritability in psychosis (Lee, DeCandia, Ripke, Yang, & Wray, 2012; Lee, Yang, et al., 2012; Purcell et al., 2009; Ripke et al., 2013; Ripke, Neale, Corvin, & Walter, 2014; Sklar, Ripke, Scott, & Andreassen, 2011). Furthermore, large‐scale genome‐wide association studies have identified more than 100 SNPs that are significantly associated with an increased risk of developing schizophrenia (Ripke et al., 2014) and bipolar disorder (Sklar et al., 2011).

However, it is still largely unknown exactly how these genetic risk variants lead to the illness, and an important goal of psychiatric genetic research is to clarify the effects and mechanisms of these variants (Carter et al., 2017; Geschwind & Flint, 2015; Glahn et al., 2014; Hall & Smoller, 2010; Harrison, 2015). Endophenotypes—biological markers that are heritable, quantitative traits associated with the illness, and observed in unaffected relatives of patients—could help us to understand the pathways from genes to the illness (Braff & Tamminga, 2017; Geschwind & Flint, 2015; Iacono, Vaidyanathan, Vrieze, & Malone, 2014; Gottesman & Gould, 2003; Meyer‐Lindenberg & Weinberger, 2006; Munafò & Flint, 2014). As endophenotypes are thought to be related to the genetic factors underlying disorders, it is likely that a subset of psychosis associated SNPs also influence them (Lencz et al., 2014; Toulopoulou et al., 2015).

The relationship between the genetics of endophenotypes and that of psychosis can be investigated using polygenic risk scores, a method that calculates the combined effect of a large number of SNPs, each with a very subtle individual effect (Purcell et al., 2009). Several studies have shown that such polygenic scores differ between patients and controls, thus providing a useful tool to measure genetic liability to psychosis in independent samples (Bramon, Pirinen, Strange, Lin, & Spencer, 2014; Derks et al., 2012; Purcell et al., 2009; Vassos et al., 2017). A number of studies have investigated the relationship between endophenotypes and polygenic risk scores for schizophrenia and bipolar disorder (Caseras, Tansey, Foley, & Linden, 2015; Hall et al., 2015; Hubbard et al., 2016; Lencz et al., 2014; Liu et al., 2017; McIntosh et al., 2013; Papiol et al., 2014; Terwisscha van Scheltinga, Bakker, van Haren, Derks, Buizer‐Voskamp, Boos, et al., 2013; Terwisscha van Scheltinga, Bakker, van Haren, Derks, Buizer‐Voskamp, Cahn, et al., 2013; Van der Auwera et al., 2015; Whalley et al., 2015, 2012, 2013). However, previous studies used a case‐control design and focused on specific endophenotypes. Here we analyse endophenotypes across three domains—anatomical, physiological and cognitive—in a large family‐based sample, including relatives who have a higher genetic load than controls but without the confounds of the illness itself.

The included measures are all putative endophenotypes for psychosis and were compatible across centers, thus reaching substantial sample sizes:

- P300 event‐related potential: reduced amplitude and prolonged latency of the P300 wave have consistently been found in patients with psychotic illnesses as well as in their unaffected relatives, compared to controls (Bestelmeyer, Phillips, Crombie, Benson, & St.Clair, 2009; Blackwood, St Clair, Muir, & Duffy, 1991; Bramon et al., 2005; Díez et al., 2013; Mondragón‐Maya et al., 2013; Pierson, Jouvent, Quintin, Perez‐Diaz, & Leboyer, 2000; Price et al., 2006; Schulze et al., 2008; Turetsky et al., 2014; Weisbrod, Hill, Niethammer, & Sauer, 1999; Winterer et al., 2003). The P300 is thought to be a correlate of attention and working memory (Ford, 2014; Näätänen, 1990).
- Cognitive performance: deficits on cognitive tests such as digit span (measuring working memory), block design (measuring working memory and spatial visualization), and the Rey Auditory Verbal Learning Task (RAVLT) immediate and delayed recall (measuring short and long term verbal memory, respectively) are common and persistent across psychotic disorders (Bora, Yucel, & Pantelis, 2009; Bora & Pantelis, 2015; Gur et al., 2007; Heinrichs & Zakzanis, 1998; Kim et al., 2015; Lee et al., 2015). Abnormalities are often observed before the onset of the illness as well as in unaffected relatives (Birkett et al., 2008; Forbes, Carrick, McIntosh, & Lawrie, 2009; Glahn et al., 2006; Ivleva et al., 2012; Park & Gooding, 2014; Reichenberg et al., 2010; Saperstein et al., 2006; Snitz, Macdonald, & Carter, 2006).
- Lateral ventricular volume: increased ventricular volume is a highly replicated finding in patients with psychosis compared to controls (Boos, Aleman, Cahn, Hulshoff Pol, & Kahn, 2007; Crespo‐Facorro et al., 2009; Fannon et al., 2000; Fusar‐Poli et al., 2013; Haijma et al., 2013; Kempton, Stahl, Williams, & DeLisi, 2010; Kumra et al., 2000; McDonald et al., 2002, 2006; Sharma et al., 1998; Shenton, Dickey, Frumin, & McCarley, 2001; Strasser et al., 2005; Wright et al., 2000). This enlargement has been attributed to neurodevelopmental impairments, psychosis progression, or the effects of antipsychotic medications (Gogtay et al., 2003; McDonald et al., 2006; Pilowsky, Kerwin, & Murray, 1993).

Past research has found a genetic overlap between these endophenotypes and psychosis. This includes a genetic correlation (overlap due to genetic factors) between the P300 amplitude and bipolar disorder (−0.33) (Hall, Rijsdijk, Kalidindi, et al., 2007) and schizophrenia (−0.48) (Hall, Rijsdijk, Picchioni, et al., 2007). Brain volume has also been shown to be genetically correlated with psychosis, including whole brain volume (−0.36) (Rijsdijk et al., 2005), and white matter volume (−0.20) (van der Schot et al., 2009). Similarly, findings indicate a genetic correlation between psychosis and cognition. Toulopoulou et al. (2007) found genetic correlations between schizophrenia and working memory (−0.79), perceptual organization (−0.61), verbal comprehension (−0.34), and IQ (−0.75), and Georgiades et al. (2016) saw genetic correlations between bipolar disorder and working memory (0.33), verbal learning (−0.46), and IQ (−0.51).

The aim of this study is to test whether polygenic risk scores for schizophrenia and bipolar disorder influence these multi‐modal psychosis endophenotypes, in a large international sample of patients with psychosis, their unaffected first‐degree relatives, and healthy controls.

## 2 METHODS

### 2.1 Sample and clinical assessment

The total sample in this study included 4,242 participants of European ancestry: 1,087 patients with psychotic illnesses (see Table 1 for breakdown of diagnoses), 822 unaffected first degree relatives of probands (with no personal history of a psychotic illness), and 2,333 unaffected controls (with no personal or family history of a psychotic illness). Relatives and controls were not excluded if they had a personal history of non‐psychotic psychiatric disorders (such as depression or anxiety), provided they were well and off psychotropic medication at the time of testing and for the preceding 12 months. This was to avoid recruiting a biased “super healthy” control group, unrepresentative of the general populations.

**Table 1:** sample characteristics (N = 4242)

	Total sample	Controls	Unaffected relatives	Patients with psychosis
Sample size (*N*, %)	4,242	2,333 (55.0%)	822 (19.4%)	1,087 (25.6%)
Age (mean years ± SD)	42.5 (±15.8)	45.7 (±16.3)	45.27 (±15.65)	33.48 (±10.39)
Age range (years)	16–85	16–84	16–85	16–79
Gender (% female)	48.5%	52.0%	60.0%	32.4%
Diagnoses (*N*)				
Schizophrenia	703	–	–	703
Bipolar I disorder	105	–	–	105
Psychosis NOS	86	–	–	86
Schizophreniform disorder	68	–	–	68
Schizoaffective disorder	60	–	–	60
Brief psychotic disorder	40	–	–	40
Other psychotic illness	25	–	–	25
Depression	273	137	136	–
Anxiety disorder	47	15	32	–
Other non‐psychotic illness	41	20	21	–
No psychiatric illness	2,794	2,161	633	–

SD, standard deviation; NOS, not otherwise specified; RAVLT, Rey auditory verbal learning task.

To confirm or rule out a DSM‐IV (APA, 1994) diagnosis, all participants underwent a structured clinical interview with either the Comprehensive Assessment of Symptoms and History (CASH; Andreasen, Flaum, & Arndt, 1992), the Structured Clinical Interview for DSM Disorders (SCID; Spitzer, Williams, Gibbon, & First, 1992), the Schedule for Affective Disorders and Schizophrenia (SADS; Endicott & Spitzer, 1978) or the Schedule for Clinical Assessment in Neuropsychiatry, Version 2.0 (SCAN; Wing et al., 1990). Participants were excluded if they had a history of neurologic disease or a loss of consciousness due to a head injury.

Recruitment occurred across 11 locations in Australia and Europe (Germany, The Netherlands, Spain, and the United Kingdom). See Supplementary Materials for a summary of the data collected from each site. Participants provided written informed consent, and the study was approved by the respective ethical committees at each of the 11 participating centers.

### 2.2 Neuropsychological assessments

The Wechsler Adult Intelligence Scale, revised version (WAIS‐R; Wechsler, 1981) or third edition (WAIS‐III; Wechsler, 1997), were administered to participants. Performance on two subtests was used for analyses: the combined forward and backward digit span (measuring attention and working memory) and block design (measuring spatial visualization). The Rey Auditory Verbal Learning Test (RAVLT; Rey, 1964), including both immediate and delayed recall (assessing short‐ and long‐term verbal memory, respectively), was also administered. Higher scores on the cognitive tasks indicate better performance. Additional information on the methodology for each site contributing data is reported elsewhere (Crespo‐Facorro et al., 2007; González‐Blanch et al., 2007; Johnstone, Ebmeier, Miller, Owens, & Lawrie, 2005; Korver, Quee, Boos, Simons, & de Haan, 2012; Toulopoulou et al., 2010; Walters et al., 2010; Waters, Price, Dragović, & Jablensky, 2009).

### 2.3 EEG data collection and processing

Electrophysiological data were obtained from three sites (Table S1). EEG data acquisition and processing varied slightly between sites and are summarized below. The full methods for each site are reported elsewhere (Bramon et al., 2005; Hall et al., 2006; Price et al., 2006; Waters et al., 2009; Weisbrod et al., 1999).

In summary, EEG was collected from 17 to 20 electrodes placed according to the International 10/20 system (Jasper, 1958). The P300 event related potential was obtained using a standard two‐tone frequency deviant auditory oddball paradigm, with standard (“non‐target”) tones of 1,000 Hz and rare (“target”) tones of 1,500 Hz. The number of tones presented varied from 150 to 800, the tones were 80 or 97 dB, lasted for 20–50 ms, and the inter‐stimulus interval was between 1 and 2 s. The majority of participants (90%) were asked to press a button in response to “target” stimuli, but a subset were asked to close their eyes and count “target” stimuli in their heads. Excluding the 10% of participants receiving different instructions does not change the results.

The data were continuously recorded in one of three ways: 500 Hz sampling rate and 0.03–120 Hz band pass filter; 200 Hz sampling rate and 0.05–30 Hz band pass filter; or 400 Hz sampling rate and 70 Hz low‐pass filter. Linked earlobes or mastoids were used as reference and vertical, and in most cases also horizontal, electro‐oculographs were recorded at each site and used to correct for eye‐blink artefacts using regression based weighting coefficients (Semlitsch, Anderer, Schuster, & Presslich, 1986). After additional manual checks, artefact‐free epochs were included and baseline corrected before averaging. The averaged waveforms to correctly detected targets were then filtered using 0.03 or 0.05 Hz high‐pass and 30 or 45 Hz low‐pass filters. The peak amplitude and latency of the P300 were measured at electrode location PZ (parietal midline), within the range of 250–550 ms post‐stimulus.

### 2.4 MRI data collection and processing

MRI data acquisition and image processing varied between sites; see previous publications and the Supplementary Materials for an outline of the methods used for each center (Collip et al., 2013; Crespo‐Facorro et al., 2009; Dutt et al., 2009; Frangou et al., 1997; Habets, Marcelis, Gronenschild, Drukker, & Van Os, 2011; Hulshoff Pol et al., 2002; Lawrie et al., 1999; Mata et al., 2009; McDonald et al., 2002; Schnack et al., 2001; Schulze et al., 2006; Steel et al., 2002; Whalley et al., 1999; Wobrock et al., 2009). Field strengths included 1, 1.5, or 3 T. Lateral ventricular volumes were measured using automatic or semi‐automatic region of interest analyses, and included the body, frontal, occipital, and temporal horns.

### 2.5 Genotyping methods

DNA was obtained from blood for all participants across each center, and sent to the Wellcome Trust Sanger Institute (Cambridge, United Kingdom). Samples were genotyped with the Genome‐wide Human SNP Array 6.0 at the Affymetrix Services Laboratory (www.affymetrix.com). We applied standard quality control procedures, phasing and imputation, and adjustments for population structure as described in the Supplementary Materials and in Bramon et al. (2014).

### 2.6 Polygenic score analysis

Following the method described in Purcell et al. (2009), polygenic risk profile scores were calculated separately for schizophrenia and bipolar disorder. Summary data from the most recent Psychiatric Genomics Consortium genome‐wide association studies for schizophrenia (PGC2) (Ripke et al., 2014) and bipolar disorder (Sklar et al., 2011) were used. In both cases, we used data from the Psychiatric Genomics Consortium that did not overlap with the sample used in the current study. For schizophrenia polygenic scores, the discovery sample included 31,658 cases and 42,022 controls (Ripke et al., 2014), and for bipolar disorder, the discovery sample included 7,481 cases and 9,250 controls (Sklar et al., 2011).

Polygenic scores for each individual were calculated using PLINK (Purcell et al., 2007), from the number of risk alleles carried for each selected SNP (i.e., 0, 1, or 2), weighted by the log(OR) provided by the Psychiatric Genomics Consortium, and averaged across all SNPs. SNPs were selected from the Psychiatric Genomics Consortium's panel using six different significance thresholds (*p*_T_ < 5 × 10^−08^, 0.001, 0.05, 0.1, 0.5, 1), hence including an increasing number of SNPs the more liberal the threshold (see Supplementary Materials for the number of SNPs included at each threshold).

### 2.7 Statistical analyses

Linear regression analyses were performed to test whether schizophrenia and/or bipolar disorder polygenic scores influence endophenotypes for psychosis. These included the P300 event related potential (amplitude and latency), lateral ventricular volume, and measures of cognition (digit span, block design, and the Rey Auditory Verbal Learning Task—RAVLT immediate and delayed recall).

Endophenotype measures were standardized for each site separately (using the overall means and standard deviations within each site) to control for differences between the centers. Covariates included in all analyses were clinical group (patient, relative, or control), study site, the first three population structure principal components, age, and gender. Because the sample included related individuals, robust standard errors were used to account for effects of clustering within families. This specifies that the standard errors allow for intragroup correlation; that is, the observations are independent between families (clusters) but not necessarily within families. The change in *R* ^2^ between a model only including the covariates and a model including covariates plus the polygenic score is reported, which represents the additional proportion of the variance explained by the polygenic risk score.

Linear regression analyses were performed for each endophenotype using the entire sample—patients with psychosis, unaffected relatives of probands, and controls—examining the associations with polygenic score at the different significance thresholds of the Psychiatric Genomics Consortium's SNP list. This was done separately for both the schizophrenia and bipolar disorder polygenic scores.

Although we tested seven endophenotypes, we know that measurements within domains are correlated (correlation matrices are presented in the Supplementary Materials) and thus a correction of *p*‐values by seven tests through Bonferroni or other methods was deemed too stringent for a hypothesis‐driven study such as this (Perneger, 1998; Rothman, 1990; Savitz & Olshan, 1995). We, therefore, corrected for three domains (EEG, MRI, cognition), with a corrected significance threshold of 0.05/3 = 0.0167, that we rounded to the slightly more stringent cut‐off of *p* < 0.01. We report the uncorrected p values for all analyses conducted throughout this study, but we highlight as significant and showing sufficient evidence of association only those surviving the correction. Statistical analyses were conducted using STATA version 13 (www.stata.com).

## 3 RESULTS

### 3.1 Sample characteristics

Demographic information and mean values of the different endophenotypes are presented in Table 1. The patient group was significantly younger compared to both relatives (mean diff. = 11.8, *p* < 0.001) and controls (mean diff. = 12.3, *p* < 0.001), whereas relatives and controls did not differ in mean age (mean diff. = 0.5, *p* = 0.4). There were more males in the patient group compared to both the control (*χ* ^2^ = 114.4, *p* < 0.001) and relative groups (*χ* ^2^ = 144.4, *p* < 0.001). The group of relatives contained more female participants than the control group (*χ* ^2^ = 15.8, *p* < 0.001). Age and gender are included as covariates in all analyses.

Mean scores on the different endophenotypes followed the expected pattern of improving performance from patients, through to relatives and controls. See Supplementary Materials for statistics of group differences and distributions across groups, after correcting for covariates of age, gender, and study site.

### 3.2 Schizophrenia polygenic risk score analysis

**Figure 1:**
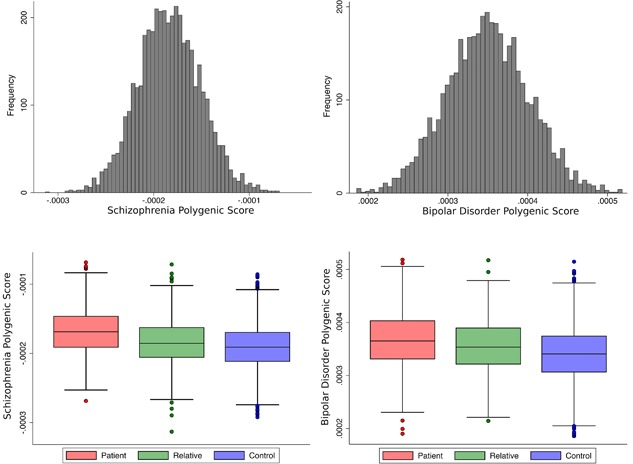
Distribution of schizophrenia (left panel) and bipolar disorder (right panel) polygenic scores at the most liberal SNP *p*‐value threshold (*p*_T_< 1), for the whole sample (upper panel) and across the three groups (lower panel) [Color figure can be viewed at wileyonlinelibrary.com]

The schizophrenia polygenic score differed significantly between the three groups (*F*(2,3184) = 86.6, *p* = 2.3 × 10^−37^; controls vs. patients *p* = 1.6 × 10^−35^, controls vs. relatives *p* = 3.6 × 10^−4^, patients vs. relatives *p* = 1.1 × 10^−16^), with patients having the highest scores, followed by relatives and lastly controls (see Figure 1, left panel).

**Figure 2:**
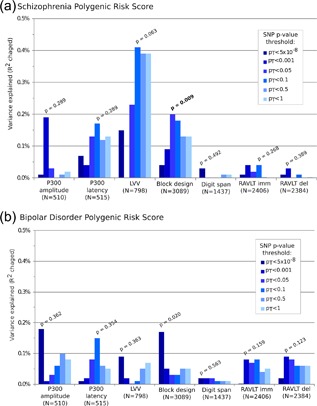
Variance explained (*R* ^2^) by schizophrenia (a) and bipolar disorder (b) polygenic scores across endophenotypes. The bars represent different single nucleotide polymorphism (SNP) *p*‐value thresholds (*p*_T_). Where a bar appears missing this is because the variance explained is too low to display given the scale used in the figure. The lowest *p*‐value for each endophenotype is displayed above the corresponding bar; the *p*‐value in bold shows a significant finding. RAVLT, Rey auditory verbal learning task; imm, immediate recall; del, delayed recall [Color figure can be viewed at wileyonlinelibrary.com]

The polygenic score for schizophrenia predicted scores on the block design task at the SNP *p*‐value threshold of *p*_T _< 0.05, with 0.20% of variance explained (*p* = 0.009). A higher polygenic score was associated with poorer performance on the block design task. The polygenic score for schizophrenia explained 0.4% of the variance in lateral ventricular volumes, the highest percentage across all phenotypes we examined; although this result was not significant (*p* = 0.063). No other associations reached significance after correcting for multiple testing. These results are shown in Figure 2a (for full results, see Supplementary Materials).

### 3.3 Bipolar disorder polygenic risk score analysis

The bipolar disorder polygenic score differed significantly between the three groups (*F*(2,3184) = 21.8, *p* = 4.0 × 10^−10^; controls vs. patients *p* = 4.9 × 10^−11^, controls vs. relatives *p* = 6.1 × 10^−4^, patients vs. relatives *p* = 2.8 × 10^−3^), with patients having the highest scores, followed by relatives and lastly controls (see Figure 1, right panel).

The polygenic score for bipolar disorder explained 0.17% of the variance in block design (at *p*_T _< 5 × 10^−8^), although this did not reach significance after correction for multiple testing (*p* = 0.02). The proportions of variances explained by the bipolar disorder polygenic score were all <0.2% (and mostly below 0.1%), and none of the other associations were significant after correcting for multiple testing. These results are shown in Figure 2b (for full results see Supplementary Materials).

### 3.4 Additional analyses

Given the fact that we are including both controls and relatives that are young enough to potentially yet develop a psychotic illness, we have repeated the analyses excluding relatives and controls under the age of 30 (202 relatives and 551 controls). This resulted in a reduction in the total sample size of 17.8% to 3489 participants. This analysis did not change the overall conclusions of the study, and has been described in the Supplementary Materials.

## 4 DISCUSSION

The aim of this study was to investigate whether polygenic risk scores for schizophrenia and bipolar disorder—based on the latest mega‐analyses from the Psychiatric Genomics Consortium—influence a range of endophenotypes for psychosis in this large international sample. This included the P300 event related potential amplitude and latency, lateral ventricular volume, and measures of cognition (block design, digit span, and the Rey Auditory Verbal Learning Task). Higher schizophrenia polygenic scores predicted poorer performance on the block design task, with 0.20% of the variance explained.

Several studies have investigated the relationship between cognition and polygenic score for schizophrenia. Terwisscha vanScheltinga, Bakker, van Haren, Derks, Buizer‐Voskamp, Cahn et al. (2013) failed to show an association with intelligence in a sample of 672 patients with schizophrenia and controls, but McIntosh et al. (2013) found an association with cognitive change between the ages of 11 and 70 in 937 controls. Further, in a large sample of 4,900 controls, Lencz et al. (2014) saw an association between schizophrenia polygenic score and general cognitive ability. Lencz et al. (2014) also calculated polygenic score for cognition (i.e., including SNPs associated with cognitive performance) in healthy controls, and used this to significantly predict disease status in a sample of over 5,000 patients with schizophrenia and 5,800 controls, with ∼0.5% of the variance in disease risk explained by the cognitive polygenic score.

Hence, research suggests that there is a genetic overlap between cognitive performance and schizophrenia susceptibility (Lencz et al., 2014; Toulopoulou et al., 2010, 2015), and our finding with the block design task is in line with this. This provides support for the notion that this measure of spatial visualization is an endophenotype for schizophrenia, and that genetic risk variants are shared between the two traits. However, there was no association between measures of working and verbal memory and this polygenic score, and furthermore, no associations reached significance for bipolar disorder polygenic score after correction for multiple testing. This could be due to a lack of power, as these genetic effects are likely to be subtle as discussed below. It is nevertheless interesting that for bipolar disorder, the association with block design approached significance at the most stringent threshold; this is the genome‐wide significant threshold and included only four SNPs that have been associated with bipolar disorder. These did not overlap with SNPs included in the schizophrenia score at this threshold, thus indicating a potential genetic overlap between block design impairment and bipolar disorder risk. Cognition has also been put forward as a possible endophenotype for bipolar disorder (Gkintoni, Pallis, Bitsios, & Giakoumaki, 2017; Miskowiak et al., 2017; Trotta, Murray, & MacCabe, 2015), although the evidence is much more limited than for schizophrenia and further research is required.

We did not find an association between polygenic risk scores for schizophrenia and bipolar disorder and the P300 event related potential. Similarly to this, studies by Hall et al. (2015) in a sample of 392 patients with schizophrenia and controls, and Liu et al. (2017) including a community‐based sample of over 4,000 individuals, both failed to show associations between the P300 and polygenic score for schizophrenia. Nevertheless, research has suggested that the P300 has a significant genetic component. Abnormalities in unaffected first‐degree relatives of patients have been identified (Schulze et al., 2008; Thaker, 2008), its heritability is around 60% (Hall et al., 2006; van Beijsterveldt & van Baal, 2002), and about 27% of variance in P300 amplitude can be accounted for by common genetic variation (Malone et al., 2014). Furthermore, a significant genetic overlap of about 34% between the P300 amplitude and bipolar disorder has been observed (Hall, Rijsdijk, Kalidindi, et al., 2007). It is possible that the overlap in common variants involved in both psychosis and the P300 is small, suggesting subtle effect sizes that are difficult to detect with very large samples required.

As for the influence of polygenic scores on measures of brain volumes, Terwisscha van Scheltinga, Bakker, van Haren, Derks, Buizer‐Voskamp, Boos, et al. (2013) and Papiol et al. (2014) both looked at total brain, white and gray matter volumes, and associations with schizophrenia polygenic score based on an early version of the Psychiatric Genomics Consortium data (including about 9,400 cases of schizophrenia); the former found a significant association whereas the latter did not. Van der Auwera et al. (2015) used data from the latest Psychiatric Genomics Consortium analysis (including nearly 37,000 patients with schizophrenia), and a test sample of 1,470 healthy controls. They failed to show an association between schizophrenia polygenic score and whole brain, gray or white matter volumes (Van der Auwera et al., 2015). Furthermore, in line with our current findings, Caseras et al. (2015) did not find an association between schizophrenia or bipolar disorder polygenic scores and lateral ventricular volume, and Franke et al. (2016) found no evidence of genetic overlap between schizophrenia and a set of subcortical brain structures.

Ventricular volume has a genetic basis with heritability estimates ranging from 30% to 70% (Carmelli, Swan, DeCarli, & Reed, 2002; Kremen et al., 2012, 2010; Peper et al., 2009; Schmitt et al., 2007). McDonald et al. (2002) found increased volumes in unaffected relatives of individuals with schizophrenia in families with more than one affected member, but not in relatives from families with only a single known case. In a meta‐analysis of 1,065 unaffected relatives of patients with and 1,100 healthy controls, Boos et al. (2007) did not find an overall effect in relatives, which is consistent with group comparisons in our sample. Enlargement of cerebral ventricles remains the best replicated biomarker in schizophrenia and bipolar disorder. The samples investigating unaffected relatives including our own are of modest size and probably have limited power to detect anatomical changes, which we would expect to be much milder than those observed among patients. Of course, the ventricular enlargement described in psychosis might also be due to illness progression, or to the effects of treatment with antipsychotic medication over time. Nevertheless, it is striking that the variance explained by the schizophrenia scores is larger for ventricular volume than for any other endophenotype we examined, with *p*‐values approaching significance, and the question remains whether with larger samples one might see an association.

Although research has shown that there is a genetic component contributing to variability in the biomarkers investigated here, these are all complex (multifactorial and heterogeneous) phenotypes, and environmental factors play important roles too. Furthermore, all endophenotypes are likely to have complex genetic influences, including a substantial polygenicity (de Geus, 2014; Geschwind & Flint, 2015; Munafò & Flint, 2014; Rees, O'Donovan, & Owen, 2015), and only a subset of SNPs associated with psychosis will also be related to particular endophenotypes, and vice versa, suggesting that effect sizes for the associations of overlapping genetic factors will be small (Lencz et al., 2014). This has indeed been found for the phenotypes investigated so far, with the amount of variance explained by polygenic scores mostly below 1% (Hall et al., 2015; Lencz et al., 2014; McIntosh et al., 2013; Papiol et al., 2014; Terwisscha van Scheltinga, Bakker, van Haren, Derks, Buizer‐Voskamp, Boos, et al., 2013; Terwisscha van Scheltinga, Bakker, van Haren, Derks, Buizer‐Voskamp, Cahn, et al., 2013; Van der Auwera et al., 2015; Whalley et al., 2015, 2012, 2013). Hence, very large samples are needed to detect such subtle effects. In future, once there are more comparable studies using similar measures, a meta‐analysis of research into associations between endophenotypes and polygenic risk scores for psychosis would be an excellent way of synthesising findings and judging where to focus future research efforts.

That the only endophenotype reaching significance (the block design task) was measured in larger samples suggests that power might indeed be an issue. For the EEG and MRI measures, that are more laborious to obtain, sample sizes in this study ranged from just over 500 to about 800, which means that a variance explained of 1–1.5% or higher could be detected, suggesting power was limited for these phenotypes. For the cognitive endophenotypes, however, the sample sizes were larger and variances explained of 0.25–0.55% or higher could be detected (see Supplementary Materials for details of this power analysis).

A limitation of this study was the heterogeneity of methods between study sites in terms of endophenotype collection, processing and analysis. This might have added noise to the data and thus reduce power to detect any true effects. However, an important strength was that genotyping of all samples was done at the same laboratory using the same platform, and that all genetic analyses and quality control were completed in a unified way. Furthermore, it is precisely through this multi‐center effort that we were able to achieve a very large sample, a key strength of this study. As the Psychiatric Genomics Consortium's work shows, large international collaborations are essential in genetic studies of common diseases and traits (Lee et al., 2013; Ripke et al., 2014; Sklar et al., 2011; Smoller et al., 2013).

Although common variants are thought to explain up to 30% of heritability in psychosis, genome wide association studies to date have only significantly identified about 3% of this (Fernandes et al., 2013; Lee, DeCandia, et al., 2012). More can be captured by calculating polygenic scores, although false positives will also be included (Iyegbe, Campbell, Butler, Ajnakina, & Sham, 2014; Wray et al., 2014). It is important to note that a larger discovery sample used to calculate polygenic scores is likely to include a higher proportion of true positive hits, and hence lead to enhanced performance of the polygenic scores as predictors of disease risk (Chatterjee et al., 2013; Dudbridge, 2013; Plomin, 2013; Wray et al., 2014). Compared to the discovery sample size used to calculate the schizophrenia polygenic score (including about 31,700 cases Ripke et al., 2014) the discovery sample for the bipolar disorder score was more than four times smaller—including only about 7,500 cases (Sklar et al., 2011)—and consequently this is the most compelling explanation of the lack of findings with the bipolar disorder polygenic score.

Importantly, there are highly significant differences in polygenic scores between the clinical groups, both in this sample and in previous studies (Bramon et al., 2014; Derks et al., 2012; Purcell et al., 2009; Ripke et al., 2014), indicating that this measure does capture genetic variants that differ between patients, unaffected relatives, and healthy controls. However, currently their predictive power is still low, and polygenic scores are not able to predict illness status accurately enough to be used in clinical practice. This would require very large discovery data sets, a large catalogue of genetic risk variants (potentially including both common and rare variants), and most likely the inclusion of a combination of genetic and non‐genetic risk factors such as cognition, brain imaging, or family history, as well as age and gender (Chatterjee et al., 2013; Dima & Breen, 2015; Dudbridge, 2013; Iyegbe et al., 2014; McCarroll & Hyman, 2013; Wray, Yang, Goddard, & Visscher, 2010).

In future, as our understanding of the genetic architecture of psychosis improves, and as discovery samples become larger, the performance of the polygenic scores is likely to be further enhanced. Polygenic scores could then be useful for testing hypotheses about the functional effects of risk variants, or to investigate the associations between disease risk and severity of illness, symptoms dimensions, and treatment or functional outcomes. This method could potentially be used to stratify populations into groups with shared genetic features, or to identify individuals at high‐risk of developing psychosis who would benefit from early therapeutic interventions (Maier et al., 2015; Wray et al., 2014). Furthermore, using polygenic scores based on selected genetic risk variants clustering on specific functional pathways, rather than a broad selection of SNPs, could become beneficial in the investigation of the specific effects that genetic risk factors for psychosis have on brain function/structure and cognition.

In conclusion, results from this large multi‐center study indicate that the combined effect of common genetic risk variants for schizophrenia is associated with spatial visualization (as measured by the block design task), providing further evidence that this measure is an endophenotype for the disorder with shared genetic risk variants. No other associations between polygenic scores for schizophrenia or bipolar disorder and endophenotypes reached significance, possibly due to a lack of power, with larger samples needed to detect these small effects. As discovery samples get larger, and additional and better targeted genetic information is included, the performance of polygenic scores will be further enhanced. Larger association studies using these scores on deeply phenotyped samples may in future provide a promising approach to investigate the effects and mechanisms of genetic risk variants for psychosis.

## CONFLICTS OF INTEREST

All authors declare that they have no financial interests or potential conflicts of interest.

## Supporting information

Additional Supporting Information may be found online in the supporting information tab for this article.

Supporting Data S1.
