# Supplementary material for: A polygenic risk score analysis of psychosis endophenotypes across brain functional, structural, and cognitive domains
Source: Am J Med Genet B Neuropsychiatr Genet. 2017 Aug 29;177(1):21–34. doi: 10.1002/ajmg.b.32581 (PMC5763362; doi:10.1002/ajmg.b.32581)
Supplement: Supplementary file 1 — Supporting Data S1. [file AJMG-177-21-s001.docx]

# Supplementary Materials

**Table S1.** Participating study centres and data collected at each site.

| **Study Site** | **Total N** | **Data contributed (N)** | | | | | | |
| --- | --- | --- | --- | --- | --- | --- | --- | --- |
|  |  | **P300 amp.** | **P300 lat.** | **LVV** | **Block Design** | **Digit Span** | **RAVLT imm. recall** | **RAVLT del. recall** |
| **Perth, Australia** | 615 | 168 | 167 |  |  |  | 615 | 615 |
| **Heidelberg, Germany** | 54 | 51 | 51 | 48 |  |  |  |  |
| **Munich, Germany** | 962 |  |  |  | 962 | 962 |  |  |
| **Edinburgh, UK** | 51 |  |  | 45 | 42 | 36 |  |  |
| **London, UK** | 656 | 291 | 297 | 318 | 480 | 258 | 6 | 6 |
| **Santander, Span** | 153 |  |  | 92 |  | 137 | 137 | 137 |
| **Pamplona, Spain** | 44 |  |  |  |  | 44 | 44 | 44 |
| **Amsterdam, The Netherlands** | 385 |  |  |  | 378 |  | 384 | 383 |
| **Groningen, The Netherlands** | 394 |  |  |  | 361 |  | 393 | 379 |
| **Maastricht, The Netherlands** | 508 |  |  | 131 | 489 |  | 496 | 496 |
| **Utrecht, The Netherlands** | 420 |  |  | 164 | 377 |  | 331 | 324 |
| ***Total N*** | *4242* | *510* | *515* | *798* | *3089* | *1437* | *2406* | *2384* |
| LVV = Lateral Ventricular Volume; RAVLT = Rey Auditory Verbal Learning Task. | | | | | | | | |

## Additional MRI methods

MRI data acquisition and image processing varied between sites and are references and outlined briefly here.

### Germany (Heidelberg)

Scanner used: 1.5 T (Tesla) Phillips. Acquisition sequence: Magnetisation prepared rapid acquisition gradient echo (MPRAGE). Acquisition protocol: Flip angle = 15°, TR = 11.4 ms, TE = 4.4 ms. Images were analysed using a region of interest tool in the software Analyze, and lateral ventricular volume was defined according to borders described in the literature (Shenton *et al.*, 2001). For full details see (Wobrock *et al.*, 2009).

### The Netherlands (Maastricht)

Scanner used: 3 T Siemens (Erlangen, Germany). Acquisition sequence: Either a modified driven equilibrium Fourier transform (MDEFT), or a magnetization prepared rapid acquisition gradient echo (MPRAGE). Acquisition protocol either; i) Flip angle = 15°, TR = 7.92 ms, TE = 2.4 ms, or ii) Flip angle = 9°, TR = 2250 ms, TE = 2.6 ms. Images were analysed using Freesurfer. Automatic labelling of each MRI voxel was carried out based on probabilistic information derived from training on a manually labelled dataset (Fischl *et al.*, 2002). For full details see (Collip *et al.*, 2013; Habets *et al.*, 2011).

### The Netherlands (Utrecht)

Scanner used: 1.5 T Philips NT. Acquisition sequence: Fast field echo (FFE). Acquisition protocol: Flip angle = 30°, TR = 30 ms, TE = 4.6 ms. Images were analysed using a Histogram method validated previously by the research group (Schnack *et al.*, 2001a). For full details see (Hulshoff Pol *et al.*, 2002; Schnack *et al.*, 2001b).

### United Kingdom (Edinburgh)

Scanner used: 1 T Siemens Magnetom (Erlangen, Germany). Acquisition sequence: Magnetisation prepared rapid acquisition gradient echo (MPRAGE). Acquisition protocol: Flip angle = 12°, repetition time (TR) = 10 ms, echo time (TE) = 4 ms. Images were analysed using a regions of interest analysis using the semi-automated programme Analyze, and lateral ventricular volume was defined by the autotrace and included frontal, occipital and temporal horns. For full details see (McIntosh *et al.*, 2004, 2005a, 2005b).

### United Kingdom (London)

Scanner used: 1.5 T General Electric (USA) Signa System. Acquisition sequence: Spoiled gradient recall (SPGR) echo. One of the following acquisition protocols was used: Flip angle = 35°, TR = 35 ms, TE = 5 ms; Flip angle = 20°, TR = 14.7 ms, TE = 3.7 ms; Flip angle = 20°, TR = 9.8 ms, TE = 2.3 ms; or Flip angle = 20°, TR = 13.1 ms, TE = 5.8 ms. Images were analysed using MEASURE, an image analysis program that uses stereologically unbiased estimation of volume. Lateral ventricular volume included the body, frontal, occipital and temporal horns, and choroid plexus where visible. For full details see (Dutt *et al.*, 2009; Frangou *et al.*, 1997; McDonald *et al.*, 2002, 2006; Schulze *et al.*, 2006).

### Spain (Santander)

Scanner used: 1.5 T General Electric Signa System (GE Medical Systems, Milwaukee, WI). Acquisition sequence: Spoiled gradient-recalled acquisition in the steady state (GRASS) (SPGR). Acquisition protocol: Flip angle = 45°, TR = 24 ms, TE = 5 ms. Images were analysed using the software BRAINS2, including automatic measurements of brain areas. For full details see (Crespo-Facorro *et al.*, 2009; Mata *et al.*, 2009).

## Genotyping details

This study includes a subset of data from a larger sample. Genotyping methods and quality control details are described in full in Bramon et al (2014) and below.

### DNA Sample Preparation

Genomic DNA obtained from blood for all participants was sent to the Wellcome Trust Sanger Institute, Cambridge, United Kingdom. Samples were processed in 96-well plate format and each plate carried a positive and a negative control. DNA concentrations were quantified using a PicoGreen assay (Invitrogen, Life Technologies, Grand Island, New York) and an aliquot assayed by agarose gel electrophoresis. A sample passed quality control if the original DNA concentration was at least 50 ng/mL and the DNA was not degraded.

### Genotyping Methodology and Quality Control

To track sample identity, 30 single nucleotide polymorphisms (SNPs) including sex chromosome markers were typed on the Sequenom platform before entry to the whole genome genotyping pipeline. Of the initial 6935 samples, 347 failed quality control due to degraded or insufficient DNA or incorrect sex classification. The remaining samples were sent for genotyping with the Genome-wide Human SNP Array 6.0 at the Affymetrix Services Lab (<http://www.affymetrix.com>).

### Data Quality Control

Genotype calling was conducted using the CHIAMO algorithm (Burton *et al.*, 2007; Marchini *et al.*, 2007) modified for use with the Affymetrix 6.0 genotyping array. A total of 11,610 SNPs with a study-wide missing data rate over 5% were excluded. Another 26,858 SNPs with four or more Mendelian inheritance errors identified with Pedstats were removed (Wigginton and Abecasis, 2005). Additional exclusion criteria were departure from Hardy-Weinberg equilibrium (p < 10^–6^) or minor allele frequency (MAF) <0.02 with 2,404 and 145,097 SNPs removed, respectively. A total of 38,895 SNPs from the X or Y chromosomes or mitochondrial DNA were also excluded from the analysis. Finally, 9,499 poorly genotyped SNPs were removed following visual inspection of the genotyping intensity plots in the program Evoker (Morris *et al.*, 2010).

214 samples were excluded with more than 2% missing data across all SNPs. Another 70 samples were excluded due to divergent genome-wide heterozygosity (inbreeding coefficients were F > 0.076 or F < -0.076 as estimated with PLINK (Purcell *et al.*, 2007). Chromosomal sharing was inferred from a genome-wide subset of 71,677 SNPs and from each duplicate pair the sample with the most complete genotype data was kept. 70 duplicates and monozygotic twins were removed by excluding one of each pair of individuals showing identity by descent greater than 95%.

Initial analysis of the genotype data identified a high fraction of samples (approximately 30%), which showed poor signal-to-noise ratio in the genotyping assay. Because the experimental source of the problem was unclear and to ensure a robust set of genotype calls, these samples were removed from further analysis. The sample loss was randomly distributed across the three clinical groups (32% of patients, 30% of relatives, and 30% of controls; χ^2^ (2 df) = 3.2; p = 0.20).

After quality control, 4,835 individuals remained. The current study included a subset of this larger sample, comprising 4,242 individuals who also had endophenotypic data available.

### Phasing and imputation

Phasing was done using Shapit2 v2.r790 (115), with default parameters except for the specification of the duoHMM flag, which allows for incorporation of known pedigree information. Imputation with reference data from the 1000 genomes panel was performed with IMPUTE2 version 2.3.0 (116; 117), using the October 2014 release of the 1000 Genome Project reference panel, and based on sequence data from 2,504 samples. Phased chromosomes were split into ~4.5 Mb chunk sizes prior to imputation, which was run with standard parameters assuming an effective population size of 20,000. After imputation, SNPs with poor imputation quality (INFO < 0.8) and missingness of > 1% were excluded.

### Population structure analysis

To investigate the genetic structure of the data, principal component analysis (PCA) of unrelated individuals was conducted using EIGENSOFT version 3.0 (118) on a thinned set of SNPs. The following SNP pruning filters were applied on 695,193 SNPs, which remained after quality control: A 10% minor allele frequency, 10^-3^ Hardy-Weinberg equilibrium deviation threshold, and all SNPs within a 1,500 SNP window had to have r^2^ below 0.2 (window shift of 150 used). Thus, a subset of 71,677 SNPs was selected for PCA using EIGENSOFT version 3.0 (118).

The first three components were included as covariates in all analyses to control for the confounding effects of population structure. This approach was used in previous work (26), and see Figure S1 for the projection of the study participants onto the first two principal components of genetic structure.

**Figure S1.** Plotted is the projection of the individuals included in this study (N=4,242) on to the first two principal components (PCs) of genetic structure. Individuals are coloured according to recruitment locations as given in legend.

**Table S2.** Number of SNPs included at each p-value threshold.

| **SNP p-value threshold (p_T_)** | **Number of SNPs** | |
| --- | --- | --- |
|  | **Schizophrenia Polygenic Score** | **Bipolar Disorder Polygenic Score** |
| p_T_ < 5x10^-8^ | 90 | 4 |
| p_T_ < 0.001 | 3,073 | 733 |
| p_T_ < 0.05 | 24,061 | 14,095 |
| p_T_ < 0.1 | 35,410 | 23,988 |
| p_T_ < 0.5 | 82,045 | 77,030 |
| p_T_ < 1 | 103,860 | 108,353 |
| SNP = Single nucleotide polymorphism | | |

## Additional Results

**Table S3.** Pairwise correlations between the endophenotypes in the whole sample including patients, relatives and controls. Shown are Pearson correlation coefficients, P-values (uncorrected for multiple testing) and the sample sizes correlations are based on.

|  | **P300 amplitude** | **P300 latency** | **LVV** | **Block Design** | **Digit Span** | **RAVLT imm. recall** |
| --- | --- | --- | --- | --- | --- | --- |
| **P300 latency** | r=-0.17  p=0.0001  N=509 |  |  |  |  |  |
| **LVV** | r=-0.01  p=0.881  N=184 | r=0.04  p=0.569  N=187 |  |  |  |  |
| **Block Design** | r=0.29  p=0.0001  N=174 | r=-0.17  p=0.020  N=180 | r=-0.11  p=0.009  N=540 |  |  |  |
| **Digit Span** | r=0.25  p=0.003  N=141 | r=-0.27  p=0.001  N=145 | r=-0.07  p=0.280  N=214 | r=0.46  p<0.0001  N=1218 |  |  |
| **RAVLT imm recall** | r=0.21  p=0.006  N=168 | r=-0.01  p=0.939  N=167 | r=-0.16  p=0.005  N=328 | r=0.30  p<0.0001  N=1555 | r=0.41  p<0.0001  N=181 |  |
| **RAVLT del recall** | r=0.22  p=0.004  N=168 | r=-0.03  p=0.709  N=167 | r=-0.16  p=0.003  N=327 | r=0.28  p<0.0001  N=1532 | r=0.38  p<0.0001  N=181 | r=0.81  p<0.0001  N=2382 |

**Table S4.** Pairwise correlations between the endophenotypes in patients only. Shown are Pearson correlation coefficients, P-values (uncorrected for multiple testing) and the sample sizes correlations are based on.

|  | **P300 amplitude** | **P300 latency** | **LVV** | **Block Design** | **Digit Span** | **RAVLT imm. recall** |
| --- | --- | --- | --- | --- | --- | --- |
| **P300 latency** | r=-0.07  p=0.310  N=210 |  |  |  |  |  |
| **LVV** | r=0.02  p=0.878  N=76 | r=-0.06  p=0.580  N=77 |  |  |  |  |
| **Block Design** | r=0.10  p=0.405  N=68 | r=-0.08  p=0.53  N=70 | r=-0.05  p=0.500  N=197 |  |  |  |
| **Digit Span** | r=0.25  p=0.085  N=49 | r=-0.36  p=0.011  N=50 | r=-0.06  p=0.485  N=129 | r=0.64  p<0.0001  N=85 |  |  |
| **RAVLT imm recall** | r=0.17  p=0.104  N=94 | r=0.16  p=0.126  N=93 | r=-0.11  p=0.194  N=143 | r=0.34  p<0.0001  N=311 | r=0.36  p<0.0001  N=173 |  |
| **RAVLT del recall** | r=0.19  p=0.071  N=94 | r=0.04  p=0.689  N=93 | r=-0.11  p=0.185  N=143 | r=0.37  p<0.0001  N=307 | r=0.35  p<0.0001  N=173 | r=0.79  p<0.0001  N=807 |

**Table S5.** Pairwise correlations between the endophenotypes in unaffected relatives only. Shown are Pearson correlation coefficients, P-values (uncorrected for multiple testing) and the sample sizes correlations are based on.

|  | **P300 amplitude** | **P300 latency** | **LVV** | **Block Design** | **Digit Span** | **RAVLT imm. recall** |
| --- | --- | --- | --- | --- | --- | --- |
| **P300 latency** | r=-0.24  p=0.002  N=160 |  |  |  |  |  |
| **LVV** | r=-0.16  p=0.178  N=71 | r=0.14  p=0.221  N=73 |  |  |  |  |
| **Block Design** | r=0.43  p=0.0003  N=68 | r=-0.18  p=0.120  N=72 | r=-0.11  p=0.181  N=143 |  |  |  |
| **Digit Span** | r=0.22  p=0.183  N=40 | r=-0.032  p=0.840  N=43 | r=-0.001  p=0.995  N=40 | r=0.77  p<0.0001  N=50 |  |  |
| **RAVLT imm recall** | r=0.05  p=0.745  N=48 | r=-0.17  p=0.260  N=48 | r=-0.03  p=0.828  N=49 | r=0.31  p<0.0001  N=470 | N=0 |  |
| **RAVLT del recall** | r=0.07  p=0.644  N=48 | r=-0.05  p=0.712  N=48 | r=-0.14  p=0.351  N=49 | r=0.28  p<0.0001  N=465 | N=0 | r=0.80  p<0.0001  N=627 |

**Table S6.** Pairwise correlations between the endophenotypes in unrelated controls only. Shown are Pearson correlation coefficients, P-values (uncorrected for multiple testing) and the sample sizes correlations are based on.

|  | **P300 amplitude** | **P300 latency** | **LVV** | **Block Design** | **Digit Span** | **RAVLT imm. recall** |
| --- | --- | --- | --- | --- | --- | --- |
| **P300 latency** | r=-0.15  p=0.071  N=139 |  |  |  |  |  |
| **LVV** | r=0.30  p=0.071  N=37 | r=-0.06  p=0.711  N=37 |  |  |  |  |
| **Block Design** | r=0.22  p=0.175  N=38 | r=0.08  p=0.649  N=38 | r=-0.13  p=0.057  N=200 |  |  |  |
| **Digit Span** | r=0.19  p=0.185  N=52 | r=-0.06  p=0.654  N=52 | r=-0.13  p=0.395  N=45 | r=0.43  p<0.0001  N=1083 |  |  |
| **RAVLT imm recall** | r=0.12  p=0.562  N=26 | r=-0.10  p=0.623  N=26 | r=-0.20  p=0.019  N=136 | r=0.30  p<0.0001  N=774 | r=0.02  p=0.974  N=7 |  |
| **RAVLT del recall** | r=0.15  p=0.455  N=26 | r=-0.14  p=0.48  N=26 | r=-0.20  p=0.021  N=135 | r=0.27  p<0.0001  N=760 | r=-0.16  p=0.725  N=7 | r=0.81  p<0.0001  N=948 |

**Table S7.** Group differences in endophenotype performance.

| **Endophenotype** | **F statistics** | **T statistics** (mean difference, p-value) | | |
| --- | --- | --- | --- | --- |
|  |  | **Controls vs patients** | **Controls vs relatives** | **Relatives vs patients** |
| **P300 amplitude** | F(2,504)=10.67, p<0.001 | 0.48, p<0.001 | 0.15, p=0.211 | 0.34; p=0.002 |
| **P300 latency** | F(2,509)=6.73, p=0.001 | -0.38, p<0.001 | -0.30, p=0.002 | -0.08, p=0.461 |
| **Lateral Ventricular Volume** | F(2,789)=1.08, p=0.344 | -0.12, p=0.142 | -0.06, p=0.571 | -0.06, p=0.524 |
| **Block Design** | F(2,3083)=54.97, p<0.001 | 0.46, p<0.001 | 0.26, p<0.001 | 0.20, p=0.001 |
| **Digit Span** | F(2,1431)=30.0 p<0.001 | 0.54, p<0.001 | 0.52, p=0.003 | 0.02, p=0.923 |
| **RAVLT imm. recall** | F(2,2400)=118.3 p<0.001 | 0.74, p<0.001 | 0.14, p=0.003 | 0.60, p<0.001 |
| **RAVLT del. recall** | F(2,2378)=92.5 p<0.001 | 0.65, p<0.001 | 0.07, p=0.172 | 0.59, p<0.001 |
| Analyses conducted on standardised scores, with study sites, participant age and gender included as covariates. RAVLT = Rey Auditory Verbal Learning Task. | | | | |

**Figure S2.** Estimated mean values of the different endophenotypes across groups (patients, relatives, controls), with 95% confidence intervals, after controlling for covariates (age, sex and study site). LVV= Lateral Ventricular Volume; RAVLT = Rey Auditory Verbal Learning Task; imm. = immediate recall; del. = delayed recall.

**Table S8.** Associations between polygenic scores and endophenotypes; full results.

| **Schizophrenia Polygenic Score** | | | | **Bipolar Disorder Polygenic Score** | | | |
| --- | --- | --- | --- | --- | --- | --- | --- |
| **P300 Amplitude (N=510)** | | | | **P300 Amplitude (N=510)** | | | |
| p_T_ | β | R^2^ | p-value | p_T_ | β | R^2^ | p-value |
| < 1 | -402.65 | 0.02% | 0.780 | < 1 | -531.35 | 0.08% | 0.514 |
| < 0.5 | -276.1 | 0.01% | 0.810 | < 0.5 | -443.863 | 0.10% | 0.454 |
| < 0.1 | 3.09 | <0.001% | 0.996 | < 0.1 | -142.56 | 0.06% | 0.588 |
| < 0.05 | -177.23 | 0.03% | 0.697 | < 0.05 | -77.52 | 0.03% | 0.678 |
| < 0.001 | -135.99 | 0.19% | 0.289 | < 0.001 | -3.413 | 0.01% | 0.783 |
| < 5×10^-08^ | 2.83 | 0.01% | 0.857 | < 5×10^-08^ | -1.824 | 0.18% | 0.362 |
| **P300 Latency (N=515)** | | | | **P300 Latency (N=515)** | | | |
| p_T_ | β | R^2^ | p-value | p_T_ | β | R^2^ | p-value |
| < 1 | 1148.7 | 0.13% | 0.382 | < 1 | -425.696 | 0.05% | 0.613 |
| < 0.5 | 912.47 | 0.12% | 0.384 | < 0.5 | -331.233 | 0.06% | 0.581 |
| < 0.1 | 561.47 | 0.17% | 0.289 | < 0.1 | -231.561 | 0.15% | 0.354 |
| < 0.05 | 384.9 | 0.13% | 0.372 | < 0.05 | -123.089 | 0.08% | 0.502 |
| < 0.001 | 59.04 | 0.04% | 0.644 | < 0.001 | -13.565 | 0.02% | 0.684 |
| < 5×10^-08^ | 9.64 | 0.07% | 0.474 | < 5×10^-08^ | -0.37 | 0.01% | 0.851 |
| **Lateral Ventricular Volume (N=795)** | | | | **Lateral Ventricular Volume (N=795)** | | | |
| p_T_ | β | R^2^ | p-value | p_T_ | β | R^2^ | p-value |
| < 1 | 1972.66 | 0.39% | 0.068 | < 1 | 531.45 | 0.07% | 0.363 |
| < 0.5 | 1576.21 | 0.39% | 0.068 | < 0.5 | 331.89 | 0.05% | 0.433 |
| < 0.1 | 849.52 | 0.41% | 0.063 | < 0.1 | 54.88 | 0.01% | 0.771 |
| < 0.05 | 490.98 | 0.23% | 0.172 | < 0.05 | -1.70 | <0.001% | 0.990 |
| < 0.001 | -2.40 | <0.001% | 0.981 | < 0.001 | 12.78 | 0.02% | 0.642 |
| < 5×10^-08^ | -13.30 | 0.15% | 0.214 | < 5×10^-08^ | -1.24 | 0.09% | 0.418 |
| **Digit Span (N=1437)** | | | | **Digit Span (N=1437)** | | | |
| p_T_ | β | R^2^ | p-value |  |  |  |  |
| < 1 | -333.5 | 0.01% | 0.678 | < 1 | 211.3 | 0.01% | 0.673 |
| < 0.5 | -232.14 | 0.01% | 0.716 | < 0.5 | 109.25 | 0.01% | 0.764 |
| < 0.1 | 37.72 | 0.001% | 0.910 | < 0.1 | 55.96 | 0.01% | 0.725 |
| < 0.05 | -24.16 | 0.001% | 0.925 | < 0.05 | -62.18 | 0.02% | 0.590 |
| < 0.001 | -2.84 | <0.001% | 0.970 | < 0.001 | -11.91 | 0.02% | 0.599 |
| < 5×10^-08^ | -6.23 | 0.03% | 0.492 | < 5×10^-08^ | -0.63 | 0.02% | 0.563 |
| **Block Design (N=3089)** | | | | **Block Design (N=3089)** | | | |
| p_T_ | β | R^2^ | p-value | p_T_ | β | R^2^ | p-value |
| < 1 | -1177.05 | 0.13% | 0.035 | < 1 | -440.98 | 0.05% | 0.226 |
| < 0.5 | -953.24 | 0.13% | 0.033 | < 0.5 | -344.74 | 0.05% | 0.190 |
| < 0.1 | -575.57 | 0.18% | 0.013 | < 0.1 | -115.82 | 0.03% | 0.309 |
| **< 0.05** | **-465.11** | **0.20%** | **0.009** | < 0.05 | -74.87 | 0.03% | 0.355 |
| < 0.001 | -86.06 | 0.09% | 0.091 | < 0.001 | 19.49 | 0.05% | 0.198 |
| < 5×10^-08^ | -6.37 | 0.04% | 0.280 | < 5×10^-08^ | 1.69 | 0.17% | 0.020 |
| **RAVLT immediate recall (N=2406)** | | | | **RAVLT immediate recall (N=2406)** | | | |
| p_T_ | β | R^2^ | p-value | p_T_ | β | R^2^ | p-value |
| < 1 | -99.8 | 0.001% | 0.866 | < 1 | 443.53 | 0.05% | 0.288 |
| < 0.5 | -137.84 | 0.003% | 0.771 | < 0.5 | 304.44 | 0.04% | 0.310 |
| < 0.1 | -271.72 | 0.04% | 0.268 | < 0.1 | 176.18 | 0.08% | 0.170 |
| < 0.05 | -147.37 | 0.02% | 0.443 | < 0.05 | 120.78 | 0.07% | 0.196 |
| < 0.001 | -54.94 | 0.04% | 0.323 | < 0.001 | 23.13 | 0.08% | 0.159 |
| < 5×10^-08^ | 4.14 | 0.01% | 0.548 | < 5×10^-08^ | -0.03 | <0.001% | 0.966 |
| **RAVLT delayed recall (N=2384)** | | | | **RAVLT delayed recall (N=2384)** | | | |
| p_T_ | β | R^2^ | p-value | p_T_ | β | R^2^ | p-value |
| < 1 | 163.2 | 0.002% | 0.788 | < 1 | 514.36 | 0.06% | 0.208 |
| < 0.5 | 98.11 | 0.001% | 0.839 | < 0.5 | 359.35 | 0.06% | 0.222 |
| < 0.1 | -116.66 | 0.01% | 0.645 | < 0.1 | 162.09 | 0.06% | 0.198 |
| < 0.05 | -39.63 | 0.001% | 0.839 | < 0.05 | 133.91 | 0.08% | 0.141 |
| < 0.001 | -48.84 | 0.03% | 0.389 | < 0.001 | 25.63 | 0.09% | 0.123 |
| < 5×10^-08^ | 2.53 | 0.01% | 0.706 | < 5×10^-08^ | -0.63 | 0.02% | 0.434 |
| pT = Single nucleotide polymorphism (SNP) p-value threshold; RAVLT = Rey Auditory Verbal Learning Task | | | | | | | |

**Table S9.** Post-hoc power calculation.

| **Endophenotype** | **N** | **Lowest R^2^ detectable** |
| --- | --- | --- |
| P300 event related potential | 515 | 1.52% |
| Lateral Ventricular Volume | 789 | 1.00% |
| Digit Span | 1437 | 0.55% |
| Rey Auditory Verbal Learning Task | 2400 | 0.32% |
| Block Design | 3089 | 0.25% |
| Alpha level 0.05, 80% power, 7 predictors in set one (covariates) and 1 predictor in set two (polygenic score).  The change in R^2^ between a model only including the covariates and a model including covariates plus the polygenic score represents the proportion of the variance explained by the polygenic risk score.  Reference: Soper DS. (2015) Post-hoc Statistical Power Calculator for Hierarchical Multiple Regression [Software] [cited 2015 Nov 25], available from: <http://www.danielsoper.com/statcalc>. | | |

### Additional analyses – Excluding controls and relatives under the age of 30

Given the fact that we are including both controls and relatives that are young enough to potentially yet develop a psychotic illness, we have repeated the analyses excluding relatives and controls under that age of 30 (202 relatives and 551 controls). This resulted in a reduction in the total sample size of 17.8% to 3489 participants. This analysis did not change the overall conclusions of the study, and has been described in the supplement.

This analysis revealed results very similar to the original findings:

For the schizophrenia polygenic risk scores, block design is nominally associated with the risk score, with the lowest p-value of p=0.0029 at the SNP p-value threshold of p_T_<0.05 and a p-value of p=0.0046 at p_T_<0.1, with 0.32% and 0.28% of variance explained, respectively. This is similar to the original analyses although the p-values have increased slightly, possible due to the reduced sample size included.

For the bipolar disorder polygenic risk score, associations with the block design at p_T_<5x10^-8^ had a p-value of p=0.0924 (R^2^=0.11%), and the lowest p-value (p=0.0603) was at p_T_<0.5 (R^2^=0.14%). This is also similar to the original findings, albeit with somewhat increased p-values.
